# Development and validation of a prognostic nomogram for predicting ventilator-associated pneumonia risk in elderly large vessel occlusion ischemic stroke after endovascular therapy patients

**DOI:** 10.3389/fnagi.2025.1654146

**Published:** 2026-01-08

**Authors:** Wenfei Liang, Jingling Zhu, Xiuling Yang, Xiaohua He, Guoshun Li, Zhaobang Chen, Jiasheng Zhao, Kangqiang Yang, Bin Liao, Huiquan Deng, Zichong Liang, Xiaoling Wu, Zhan Zhao, Weimin Ning, Qiuxing He, Jingyi Chen

**Affiliations:** 1 Department of Neurology, Dongguan Hospital of Guangzhou University of Chinese Medicine, Dongguan, China; 2 Dongguan Key Laboratory of Intractable Brain Diseases in Dongguan, Dongguan Hospital of Guangzhou University of Chinese Medicine, Dongguan, China; 3 State Key Laboratory of Dampness Syndrome of Chinese Medicine, Dongguan Hospital of Guangzhou University of Chinese Medicine, Dongguan, China

**Keywords:** elderly stroke patients, acute ischemic stroke with large vessel occlusion, ventilator-associated pneumonia, endovascular treatment, nomogram

## Abstract

**Background:**

Acute ischemic stroke with large vessel occlusion (AIS-LVO) poses a grave threat to the health of the elderly, exhibiting a high degree of disability and mortality. Post-stroke ventilator-associated pneumonia (VAP) significantly impairs neurological recovery and worsens clinical outcomes. This study aimed to construct and validate a prognostic nomogram to forecast VAP risk in elderly patients who underwent endovascular therapy (EVT) with AIS-LVO.

**Methods:**

We retrospectively analyzed a total of 536 patients with AIS-LVO who endured EVT under mechanical ventilation at the Dongguan Hospital of Guangzhou University of Chinese Medicine from August 2018 to March 2025. After applying inclusion/exclusion criteria, 240 elderly patients were randomly split into two groups: training (*n* = 168) and validation (*n* = 72), maintaining a 7:3 ratio. Using the least absolute shrinkage and selection operator regression (LASSO) for feature selection followed by multivariable logistic regression, we identified independent predictors for nomogram construction. Model performance was assessed through the area under receiver operating characteristic (ROC), calibration curves, decision curve analysis (DCA), and clinical impact curves (CIC).

**Results:**

Six independent predictors were identified: gender (OR 0.34, 95% CI 0.13∼0.85), nasogastric intubation (OR 7.56, 95% CI 1.77∼32.25), postoperative platelet-to-lymphocyte ratio(PLR) (OR 1.01, 95% CI 1.01∼1.02), postoperative neutrophil-to-lymphocyte ratio (NLR) (OR 1.22, 95% CI 1.02∼1.45), admission white blood cell(WBC) (OR 1.25, 95% CI 1.04∼1.49)and prognostic nutritional index (PNI) (OR 0.85, 95% 0.79∼0.92). The nomogram demonstrated excellent discrimination (AUROC 0.880, 95% CI 0.826∼0.933) and good calibration. DCA and CIC confirmed clinical utility across a wide probability threshold range.

**Conclusion:**

We developed and validated an effective nomogram incorporating six clinically accessible parameters to forecast VAP risk in elderly stroke patients post-EVT. This tool has the potential to expedite early high-risk patient identification and conduct preventive measures to enhance patient clinical outcomes.

## 1 Introduction

Acute ischemic stroke with large vessel occlusion (AIS-LVO) poses a grave threat to the elderly health exhibiting a high degree of disability and mortality. Endovascular therapy (EVT) has been a recommended therapy for AIS-LVO patients to achieve revascularization and improve prognosis (Goyal et al., 2015). A Chinese study demonstrated that ventilator-associated pneumonia (VAP) incidence could reach 48.4% (Zhang et al., 2015), with a mortality rate of up to 43.2% (Lin et al., 2011; Xie et al., 2011). Furthermore, stroke patients who underwent delayed extubation (more than 24 h) following mechanical thrombectomy exhibited a greater pneumonia occurrence compared to those extubated within 24 h (Saber et al., 2021). This study categorizes individuals aged 60 and above as elderly patients. The age of 60 years, widely utilized by the World Health Organization (WHO), the United Nations (UN), and in global demographic reporting, serves as the most common lower boundary for defining an “older person” internationally. This threshold is widely used in global reports, action plans, and demographic projections (Amuthavalli Thiyagarajan et al., 2022; Chatterji et al., 2015; Officer et al., 2016; Ofori-Asenso et al., 2019). Moreover, elderly AIS-LVO patients often present with critical illnesses and poor baseline conditions, predisposing them to common postoperative complications. The frequent need for mechanical ventilation to manage these critical states further elevates the risk of VAP. Additionally, a high prevalence of pneumonia is linked to several stroke-related impairments, including dysphagia from brainstem involvement, consciousness disorders, and stroke-induced immunosuppression (SIIS)—a condition that heightens susceptibility to bacterial infections (Hannawi et al., 2013). Furthermore, prolonged hospitalization after EVT increases exposure to pathogenic bacteria, resulting in elevated infection rates. It is noted that pneumonia constitutes an independent influence on the poor functional outcome of stroke patients. The inflammatory response associated with VAP can aggravate post-stroke brain injury, possibly resulting in serious consequences such as sepsis, multi-organ failure, septic shock, gastrointestinal hemorrhage, and even death (Ji et al., 2013). As for elderly patients with AIS-LVO, their advanced age, comorbidities, and specific immune and inflammatory responses distinguish them from the rest of the critically ill population, leading to different VAP risk profiles and outcomes (Liu and Guo, 2018; Wang et al., 2021; Xu et al., 2019).

Ventilator-associated pneumonia represents a distinct subtype of pneumonia that poses a particular clinical challenge, owing to its specific pathophysiological features and diagnostic complexities. It is specifically defined as a healthcare-associated pulmonary infection in patients who have undergone invasive mechanical ventilation (tracheal intubation/tracheotomy) for over 48 h or within 48 h of extubation (Papazian et al., 2020). The risk of developing VAP peaks around the fifth day of mechanical ventilation (Kalanuria et al., 2014). However, stroke-associated pneumonia (SAP), as a diagnostic term proposed by the Stroke Pneumonia Consensus Group in 2015, specifically refers to pneumonia complicating stroke patients not receiving mechanical ventilation within 7 days of the onset of stroke (Smith et al., 2015). Consequently, lung infections in stroke patients mechanically ventilated after EVT should be classified as VAP. However, the current diagnostic paradigm often fails to clearly distinguish VAP from other pneumonias. This difficulty arises from overlapping clinical manifestations and non-specific diagnostic criteria, such as radiographic infiltrates, fever, and leukocytosis. Diagnostic uncertainty often delays VAP identification, leading to delayed or inappropriate therapy. Early recognition and intervention of VAP are crucial for the subsequent treatment of patients and the enhancement of their prognoses, and thus, clinicians should pay close attention.

Several studies have explored the factors associated with VAP incidence in the elderly, such as the underlying diseases, inflammation biomarkers, and the mechanical ventilation duration (Liu and Guo, 2018; Wang et al., 2021; Xu et al., 2019). However, there is a paucity of research focusing on VAP occurrence in this specific group of elderly AIS-LVO patients after EVT. Furthermore, the majority of extant studies are limited to single-factor analyses, failing to delve into the intricate interplay among multiple risk factors.

The nomogram is a visual prediction model that integrates multiple risk factors, providing clinicians with an intuitive and individualized tool for predicting various diseases. However, there is a paucity of relevant nomograms that have been reported for the prediction of post-interventional VAP in elderly AIS-LVO patients. Our research team successfully constructed a prediction model of VAP in AIS-LVO patients after EVT using single-center cohort data (*n* = 184), and its efficacy in predicting VAP was demonstrated (AUROC = 0.880) (Zhu et al., 2024). Based on preceding studies, this study incorporated three systematic methodological enhancements to account for sample size restrictions and population generalizability challenges. Firstly, expanding the sample size and focusing on the elderly (>60 years) improved the research cohort’s relevance and clinical representativeness. Secondly, the development of the model utilized a training and validation cohort, enhancing the robustness and reliability of model results through hierarchical validation of the data. Finally, this research introduced CIC to validate the model’s predictive performance.

This study aimed to construct a dynamic assessment system for predicting postoperative VAP risk in elderly AIS-LVO patients. The model’s potential may be further enhanced by exploring independent predictors with therapeutic intervention significance. Ultimately, this would provide a solid evidence-based foundation for the development of individualized VAP prevention strategies in clinical practice.

## 2 Materials and methods

### 2.1 Study design and patient selection

We conducted a retrospective cohort analysis involving patients with AIS-LVO who underwent EVT at Dongguan Hospital of Guangzhou University of Chinese Medicine (August 2018 to March 2025). The inclusion criteria for mechanically thrombectomy patients in this study strictly adhered to the standard protocol, primarily referencing the enrollment criteria from the DAWN trial and DEFUSE-3 trial, as well as the Chinese Guidelines for Endovascular Treatment of Acute Ischemic Stroke (2023 Edition) (Albers et al., 2018; Chinese Stroke Society et al., 2023; Nogueira et al., 2018). The specific criteria are as follows:(1) Onset time: Within 24 h of last known normal status; (2) Age: ≥18 years old (all patients in this study were ≥60 years old); (3) Neurological deficit severity: National Institutes of Health Stroke Scale (NIHSS) score ≥ 6; (4) Core infarct volume: Alberta Stroke Project Early CT score (ASPECTS) ≥ 6; (5) Presence of imaging-clinical mismatch or perfusion-core mismatch (patients preoperative mRS scores and ASPECTS scores in Supplementary Material 1).

Regarding the 90-day follow-up, we completed follow-ups for all enrolled patients through a combination of telephone calls and outpatient clinic visits. Specifically, 90 days after endovascular thrombectomy (EVT), we collected information on patient prognosis via a standardized telephone follow-up protocol. Where questions arose or data were unclear during the telephone follow-up, we arranged additional outpatient clinic visits to verify the information.

The subsequent inclusion criteria were delineated: (1) Age ≥ 60 years; (2) Confirmed LVO (internal carotid artery, M1/M2 middle cerebral artery, or basilar artery) by computed tomography angiography (CTA), magnetic resonance angiography (MRA), or digital subtraction angiography (DSA); (3) AIS diagnosis according to World Health Organization criteria with neuroimaging confirmation [computed tomography (CT) or magnetic resonance imaging (MRI)]; (4) Received EVT with mechanical ventilation. Exclusion criteria:(1) Age < 60 years; (2) Patients complicated with severe primary diseases such as vital organ failure and hematopoietic system diseases, or severe infections; (3) Patients with incomplete medical records; (4) Patients who refused follow-up or could not be contacted. After screening 536 patients, 240 met eligibility criteria and were subsequently allocated into a training cohort (*n* = 168, 70%) and a validation cohort (*n* = 72, 30%) in a 7:3 ratio. This research was approved by the Institutional Review Board of Dongguan Hospital (No. PJ [2025] 87). Informed consent was exempted because the research utilized retrospectively collected anonymized data.

### 2.2 Clinical outcomes

The incidence of VAP following EVT in AIS-LVO was designated as the principal endpoint of this investigation. Referring to the diagnostic criteria for VAP (Smith et al., 2015; Spalding et al., 2017), VAP is defined as pneumonia occurring 48 h after tracheal intubation or tracheostomy with mechanical ventilation, or within 48 h after weaning from mechanical ventilation and extubation. The clinical diagnosis of VAP is confirmed when chest imaging, including X-rays or CT scans, reveals novel or worsening infiltrates, consolidations, or ground-glass opacities, and at least two of the following four clinical symptoms are present: (1) Temperature > 38 °C; (2) The presence of purulent sputum; (3) Abnormal white blood cell counts (either > 10 × 10^9^/L or < 4 × 10^9^/L); (4) Respiratory distress symptoms such as coughing, shortness of breath, or rapid breathing (respiratory rate > 25 breaths/min). VAP was diagnosed by two independent neurologists blinded to predictor variables. Additionally, postoperative complications include symptomatic intracranial hemorrhage (sICH), gastrointestinal hemorrhage, and brain herniation. sICH is defined as an intracerebral hemorrhage detected on neuroimaging accompanied by significant clinical deterioration, most commonly measured as an increase of ≥4 points on the NIHSS, or resulting in death. The European Cooperative Acute Stroke Study (ECASS) standard was adopted, and detailed bleeding subtype analysis data are provided in “Supplementary Material 1,” including patient distribution by subtype (HI-1, HI-2, PH-1, PH-2).

**FIGURE 1:**
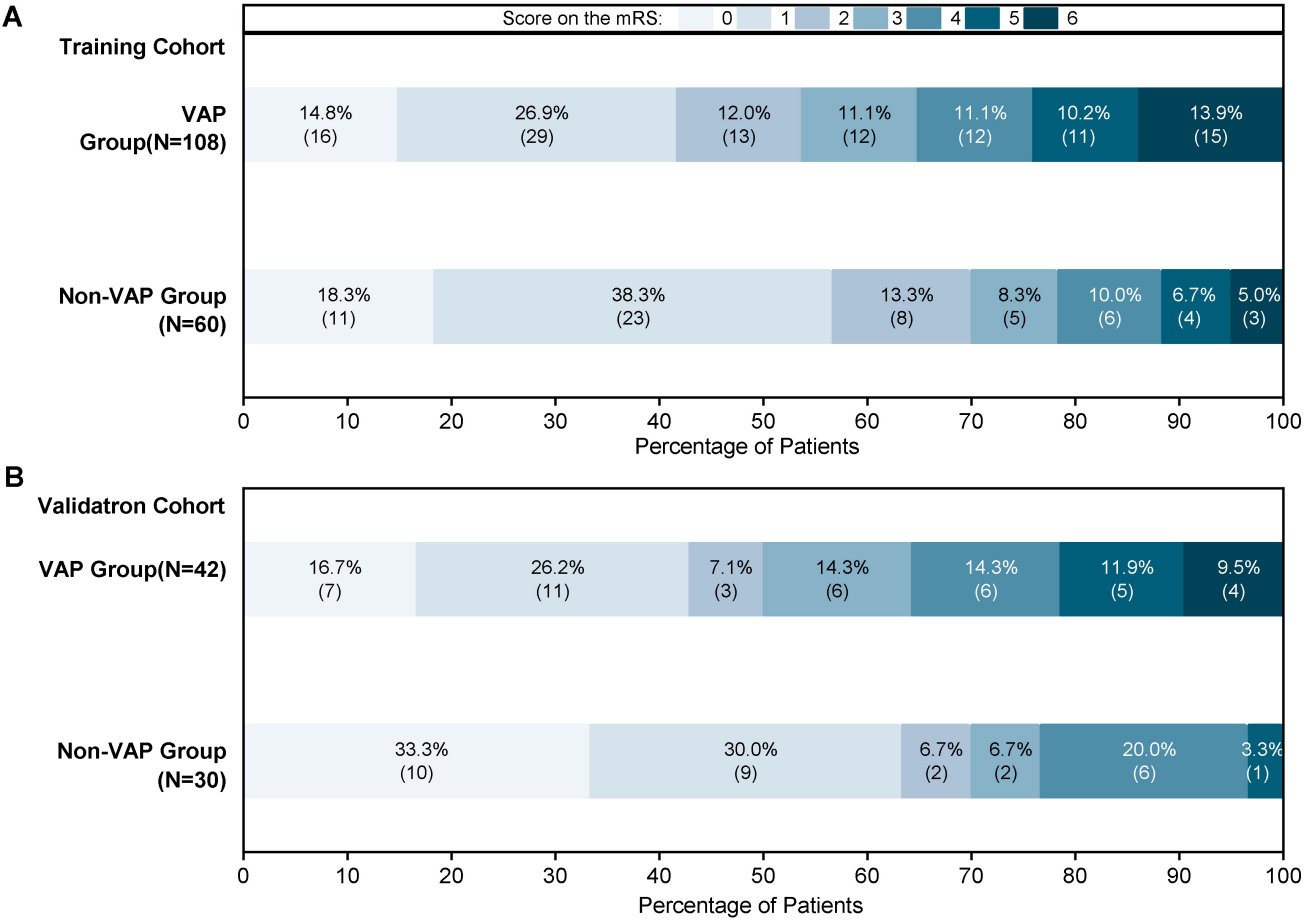
Proportions of ventilator-associated pneumonia (VAP) and non-VAP patients according to the 90-mRS results. **(A)** The 90-mRS training cohort. **(B)** The 90-mRS validation cohort. A total of 240 patients were incorporated. Clinical results of individuals with VAP and non-VAP were monitored three months post-endovascular therapy (EVT).

The secondary outcomes were 90-day prognostic outcomes, including 90-day mortality and a modified Rankin Scale (mRS) score (Figure 1). An mRS score within the 0–2 range was regarded as a good outcome, and scores within the 3–6 range were classified as having a negative outcome.

### 2.3 Predictor factor selection

Through analysis of existing literature and clinical experience, 39 predictive variables for VAP in post-stroke patients who underwent EVT were identified. Variables were systematically extracted from electronic medical records and categorized as follows: (1) Demographics: gender, age; (2) Medical history: diabetes, hypertension, atrial fibrillation (AF), prior stroke, heart failure (HF), chronic obstructive pulmonary disease (COPD), coronary artery disease (CAD), smoking status, alcohol drinking; (3) Clinical characteristics: Disturbance of consciousness, Systolic/diastolic admission blood pressure, dysphagia (Kubota water swallow test ≤ II), admission NIHSS, Glasgow Coma Scale (CGS), modified Rankin Scale (mRS) score(0–2score); (4) Laboratory results: HbA1 (Hemoglobin A1), Admission Glu ≥ 11.0, albumin (ALB), prognostic nutritional index (PNI), triglyceride-glucose index (TyG), admission white blood cell count(WBC), postoperative C-reactive protein (CRP), postoperative systemic immune-inflammatory index (SII), postoperative systemic inflammatory response index (SIRI), postoperative neutrophil-to-lymphocyte ratio (NLR), postoperative platelet-to-lymphocyte ratio (PLR); stroke location (anterior circulation, posterior circulation); leukoencephalopathy severity (measured by Fazekas scale: 0–3 points corresponding to none–mild–moderate–severe), TOAST classification;(5) Clinical procedures: Operation time, modified Thrombolysis in Cerebral Infarction (mTICI) score (2b/3 vs. <2b), presence of nasogastric tube and nasogastric tube resection within 7 days, duration of mechanical ventilation and length of stay in the intensive care unit (ICU).

### 2.4 Statistical analysis

Data analysis and visualization were employed SPSS 27.0, the R program (Version 4.2.3), and Origin 2024. A descriptive statistical analysis was performed on 240 participants. For continuous variables, normality was assessed using the Shapiro-Wilk test combined with histograms. They were reported as mean ± SD if normally distributed (*P* > 0.05) or median (IQR) if non-normally distributed (*P* ≤ 0.05). Intergroup differences for continuous variables were analyzed using the independent samples *t*-test (for normally distributed data) or nonparametric Mann-Whitney U test (for non-normally distributed data). Categorical variables were characterized by frequencies and percentages (%), and differences were evaluated using the chi-square test or Fisher’s exact test. A two-sided *P* < 0.05 was considered statistically significant.

The nomogram was created using a three-stage framework: (1) Predictor Screening: a two-stage selection approach was employed. LASSO regression with 10-fold cross-validation mitigated multicollinearity and overfitting. Standardized variables established the best lambda for identifying meaningful predictors. The chosen factors were further evaluated by multivariable logistic regression to ascertain their independent prognostic significance. (2) Nomogram Construction: variables deemed significant in both LASSO and multivariable logistic regression were utilized to construct the nomogram, with weights allocated according to regression coefficients. (3) Clinical Validation: the model’s performance was assessed using the receiver operating characteristic (ROC) curve, with an area under the curve (AUROC) > 0.7 signifying robust discriminative capability. Calibration curves, decision curve analysis (DCA), and clinical impact curves (CIC) were utilized to assess the nomogram’s clinical relevance and net benefit at different probability thresholds. Additionally, to verify the adequacy of sample size for detecting intergroup differences, a post-hoc power analysis was performed using G*Power 3.1 software (Supplementary Material 2).

## 3 Results

### 3.1 Flowchart of study

**FIGURE 2:**
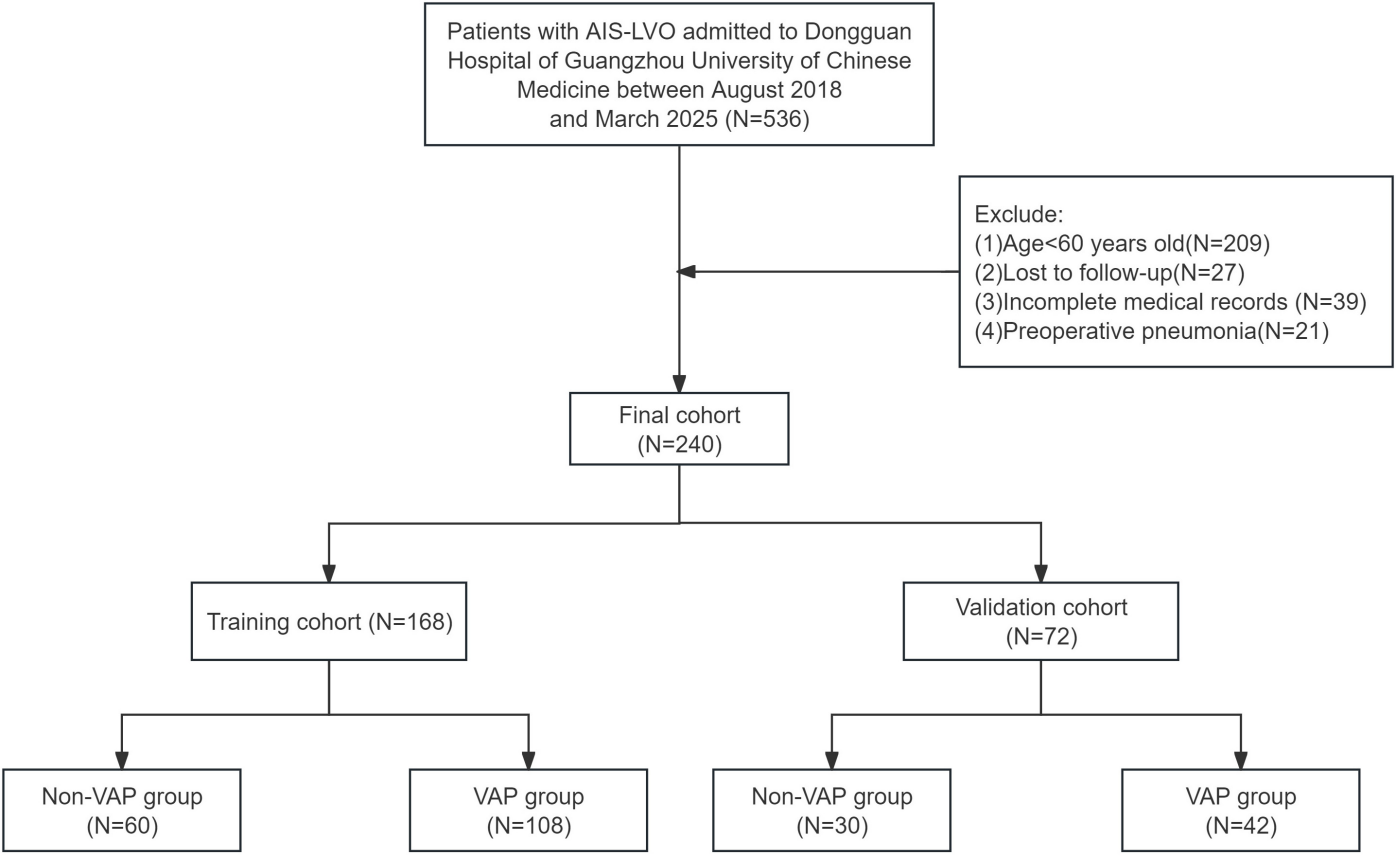
Study design flowchart.

Figure 2 presents the participant selection flowchart. From an initial cohort of 536 patients with AIS-LVO who underwent EVT, 296 were excluded based on predefined criteria (detailed in section “2 Materials and methods”). The final analytical cohort comprised 240 patients, who were allocated to either the training cohort (*n* = 168, 70%) or validation cohort (*n* = 72, 30%) in a 7:3 ratio.

### 3.2 Patient characteristics

This study included 240 patients, categorized into two groups: 90 patients without VAP (37.5%) and 150 VAP patients (62.5%). To ensure the reliability of the study, 240 patients were allocated into training (*n* = 168) and validation (*n* = 72) cohorts (7:3 ratio). The baseline data of each group were statistically analyzed, respectively. Table 1 shows the differences in demographic characteristics, medical history, clinical features, inspection results, postoperative complications, procedure-related, and prognosis between the VAP and non-VAP groups (Table 1).

**TABLE 1:** A comparison of the patient characteristics between the training cohort and validation cohort.

Variables		Training cohort			Validation cohort		
		Non-VAP group (*n* = 60)	VAP group (*n* = 108)	*P*-value	Non-Vap Group (*n* = 30)	VAP group (*n* = 42)	*P-*value
**Demographics**							
Gender, male, *n* (%)		44 (73.3%)	62 (57.4%)	0.040	20 (66.7%)	27 (64.3%)	0.834
Age, years, median (IQR)		72.47 ± 9.11	74.44 ± 8.28	0.154	71.97 ± 8.13	70.76 ± 8.62	0.551
**Medical history**							
Hypertension, *n* (%)		38 (63.6%)	81 (75.0%)	0.111	18 (60.0%)	26 (61.9%)	0.870
Diabetes, *n* (%)		21 (35.0%)	34 (31.5%)	0.641	7 (23.3%)	10 (23.8%)	0.963
Atrial fibrillation, *n* (%)		17 (28.3%)	27 (25.0%)	0.638	3 (10.0%)	7 (16.7%)	0.645
Heart failure		4 (6.7%)	6 (5.6%)	0.772	1 (3.3%)	0 (0.0%)	0.865
Prior stroke, *n* (%)		13 (21.7%)	23 (21.3%)	0.955	6 (20.0%)	13 (31.0%)	0.299
CAD, *n* (%)		9 (15.0%)	20 (18.5%)	0.563	2 (6.7%)	5 (11.9%)	0.737
COPD, *n* (%)		2 (3.3%)	3 (2.8%)	0.840	3 (10.0%)	1 (2.4%)	0.384
Smoking status, *n* (%)		19 (31.7%)	32 (29.6%)	0.783	11 (36.7%)	14 (33.3%)	0.770
Alcohol drinking, *n* (%)		12 (20.0%)	16 (14.8%)	0.388	5 (16.7%)	8 (19.0%)	0.796
**Clinical features**							
Dysphagia, *n* (%)		25 (41.7%)	40 (37.0%)	0.555	10 (33.3%)	23 (54.8%)	0.072
Disturbance of consciousness, *n* (%)		21 (35.0%)	40 (37.0%)	0.792	8 (26.7%)	19 (45.2%)	0.109
Systolic pressure, mmHg, mean ± SD		151.78 ± 25.09	152.85 ± 22.79	0.779	150.67 ± 23.24	148.12 ± 22.79	0.644
Diastolic pressure, mmHg, mean ± SD		86.73 ± 14.44	87.34 ± 13.59	0.786	86.17 ± 14.8	86.43 ± 9.92	0.929
Admission NIHSS, median (IQR)		10.00 (6.00–13.00)	9.00 (4.00–15.00)	0.702	6.0 (3.00–13.00)	12.00 (6.00–17.00)	0.030
Admission GCS, median (IQR)		15.00 (13.00–15.00)	15.00 (11.00–15.00)	0.339	15 (13.75–15)	14.5 (11–15)	0.179
Admission mRS, 0∼2 score, *n* (%)		11 (18.3%)	21 (19.4%)	0.861	11 (36.7%)	6 (14.3%)	0.027
**Laboratory results**							
Admission Glu ≥ 11.0 mmol/L, *n* (%)		7 (11.7%)	13 (12.0%)	0.943	3 (10.0%)	1 (2.4%)	0.384
HbA1, %, median (IQR)		6.10 (5.60–7.33)	5.95 (5.60–6.80)	0.333	5.9 (5.48–6.2)	5.8 (5.3–6.6)	0.627
TyG, median (IQR)		9.53 ± 0.74	9.63 ± 0.58	0.371	9.64 ± 0.54	9.36 ± 0.59	0.047
Albumin, mmol/L, mean ± SD		38.56 (35.73–41.03)	37.05 (34.48–39.75)	0.017	37.6 (35.8–40.73)	37.85 (34.23–39.80)	0.309
PNI, median (IQR)		47.83 (43.11–52.36)	43.95 (40.10–47.86)	<0.001	46.5 (42.3–50.5)	45.87 (42.15–49.51)	0.530
Admission WBC, 10^9^/L, median (IQR)		7.86 (6.63–9.35)	8.45 (7.31–11.67)	0.007	8.56 (7.31–10.24)	9.29 (7.37–11.13)	0.288
Postoperative CRP, mg/L, median (IQR)		4.40 (1.80–10.85)	6.00 (2.60–19.98)	0.066	3.50 (2.18–12.63)	5.85 (2.23–39.68)	0.158
Postoperative NLR, median (IQR)		4.07 (2.45–5.66)	7.61 (4.47–11.12)	<0.001	5.04 (2.79–8.13)	9.62 (6.30–15.43)	<0.001
Postoperative SII, median (IQR)		799.81 (517.14–1251.61)	1641.55 (1077.74–2554.48)	<0.001	977.53 (622.61–1585.91)	2189.91 (1215.38–3503.91)	<0.001
Postoperative PLR, median (IQR)		118.21 (88.67–156.87)	199.53 (150.95–277.79)	<0.001	138.15 (91.87–213.14)	259.07 (157.92–350.62)	<0.001
Postoperative SIRI, median (IQR)		1.60 (0.96–2.58)	2.36 (1.61–5.03)	<0.001	2.08 (0.95–3.40)	2.45 (1.26–5.08)	0.242
Anterior circulation, *n* (%)		50 (83.3%)	89 (82.4%)	0.879	24 (80.0%)	35 (83.3%)	0.717
Fazekas	0	9 (15.0%)	10 (9.3%)	0.151	5 (16.7%)	7 (16.7%)	0.690
	1	34 (56.7%)	57 (52.8%)	–	12 (40.0%)	22 (52.4%)	–
	2	13 (21.7%)	32 (29.6%)	–	8 (26.7%)	7 (16.7%)	–
	3	4 (6.7%)	9 (8.3%)	–	5 (16.7%)	6 (14.3%)	–
TOAST	LAA	44 (73.3%)	76 (70.4%)	0.843	27 (90.0%)	28 (66.7%)	0.070
	CE	11 (18.3%)	22 (20.4%)	–	2 (6.7%)	8 (19.0%)	–
	ODC	2 (3.3%)	2 (1.2%)	–	0 (0.0%)	3 (7.1%)	–
	SAO	3 (5.0%)	8 (7.4%)	–	1 (3.3%)	3 (7.1%)	–
Postoperative complications		–	–	0.238	–	–	0.882
Symptomatic intracranial hemorrhage, *n* (%)		13 (21.7%)	18 (16.7%)	–	5 (16.7%)	8 (19.0%)	–
Gastrointestinal hemorrhage, *n* (%)		1 (1.7%)	0 (0.0%)	–	1 (3.3%)	1 (2.4%)	–
Brain herniation, *n* (%)		1 (1.7%)	6 (5.6%)	–	1 (3.3%)	3 (7.1%)	–
**Procedure-related**							
Operation time, hours, median (IQR)		1.99 (1.35–2.39)	1.83 (1.34–2.57)	0.776	2.00 (1.58–2.27)	1.87 (1.43–2.27)	0.623
mTICI ≥ 2b, *n* (%)		57 (95%)	101 (93.5%)	0.961	29 (96.7%)	40 (95.2%)	0.762
Nasogastric intubation, *n* (%)		51 (85.0%)	101 (93.5%)	0.071	26 (86.7%)	39 (92.9%)	0.638
Reset Nasogastric tube within 7 days, *n* (%)		15 (25.0%)	46 (42.6%)	0.023	7 (23.3%)	19 (45.2%)	0.056
Duration of mechanical ventilation, hours, median (IQR)		19.13 (12.20–34.78)	20.74 (14.60–69.38)	0.054	17.4 (12.23–38.42)	20.56 (12.39–65.92)	0.093
ICU, day, median (IQR)		0.73 (0.59–1.36)	1.15 (0.69–2.38)	0.003	0.73 (0.58–1.49)	1.06 (0.65–2.36)	0.086
**Prognosis**							
90d mRS, 0∼2 score, *n* (%)		42 (70%)	58 (53.7%)	0.039	21 (70.0%)	21 (50.0%)	0.090
Mortality, *n* (%)		3 (5.0%)	15 (13.9%)	0.074	0 (0.0%)	4 (9.5%)	0.223

IQR, interquartile range; CAD, coronary artery disease; COPD, chronic obstructive pulmonary disease; NIHSS, National Institute of Health Stroke Scale; GCS, Glasgow Coma Scale; mRS, modified Rankin Scale; HbA1, Hemoglobin A1; TyG, Triglyceride-Glucose = ln[TG(mg/dl) × FPG(mg/dl)/2]; PNI, Prognostic Nutritional Index = Serum Albumin (g/L) + 5 × Peripheral Blood Lymphocyte Count (10^9^/L); WBC, White blood cell; CRP, C-reactive protein; NLR, Neutrophil-to-Lymphocyte Ratio = Neutrophil count/Lymphocyte count; SII, Systemic Immune Inflammatory Index = (Neutrophil count × Platelet count)/Lymphocyte count; PLR, Platelet-to-Lymphocyte Ratio = Platelet count/Lymphocyte count; SIRI, Systemic Inflammatory Response Index = (Neutrophil count × Monocyte count)/Lymphocyte count; TOAST, Trial of Org 10172 in Acute Stroke Treatment; LAA, large-artery atherosclerosis; CE, cardioembolism; ODC, stroke of other determined cause; SAO, small-artery occlusion.

### 3.3 Nomogram construction

#### 3.3.1 Independent risk factors in the training cohort

**FIGURE 3:**
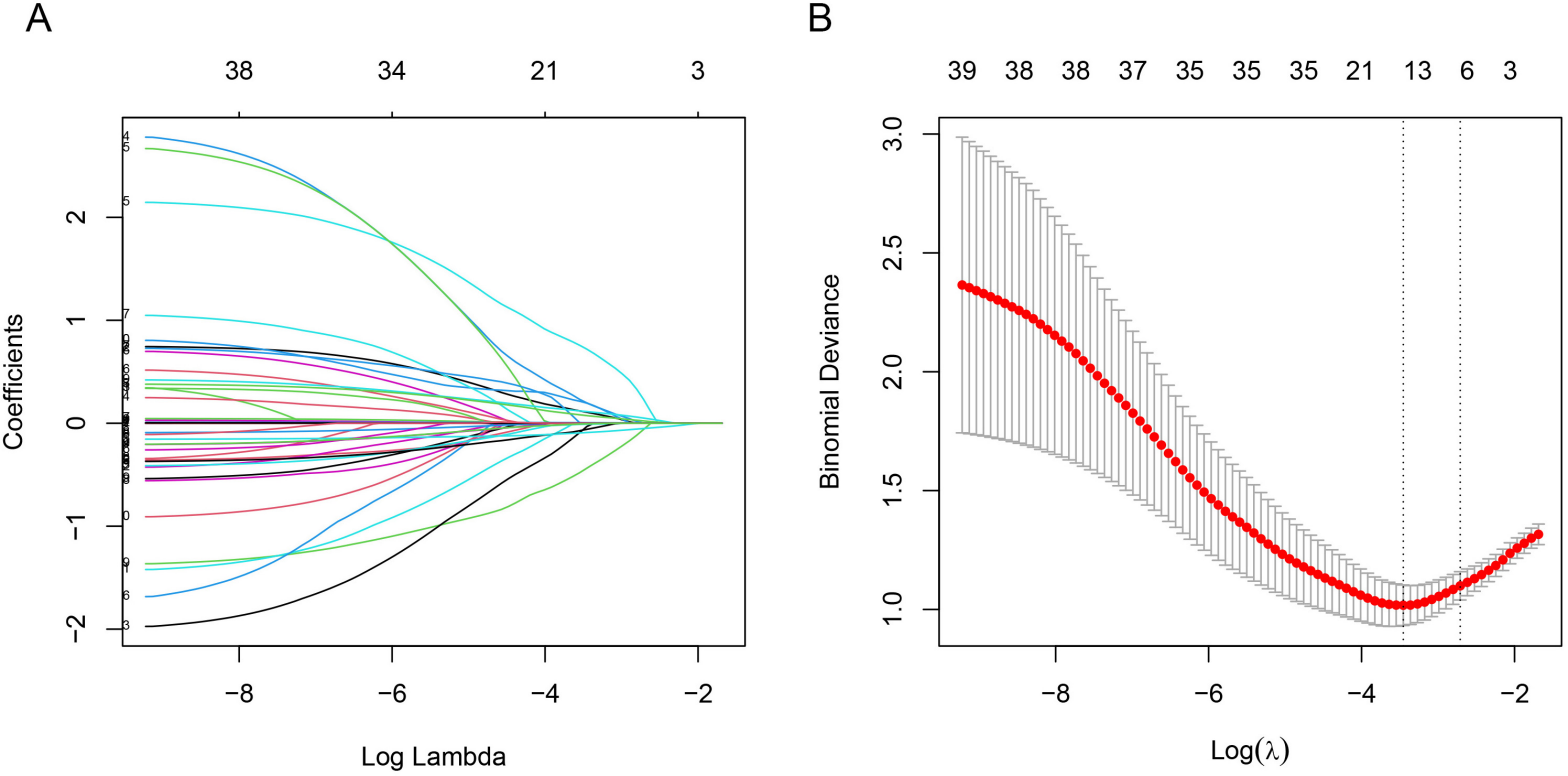
Illustrates the least absolute shrink age and selection operator regression (LASSO) variable selection process. **(A)** Coefficient profile plot: The LASSO coefficients of 39 candidate features are plotted against the log(λ) sequence. As the log(λ) value increases, the coefficients of the features gradually shrink toward zero. When the optimal lambda value (lambda min, λ = 0.032) was applied, seven variables with non-zero coefficients were selected for further analysis. **(B)** 10-fold cross-validation plot: The x-axis represents the log(λ) of the optimal parameter, while the y-axis denotes the binomial deviance (an indicator for evaluating model performance). The vertical dotted line in the plot marks the λ value derived from 10-fold cross-validation, which corresponds to lambda min (λ = 0.032) that minimizes the binomial deviance.

Least absolute shrink age and selection operator regression was employed to screen 39 variables for predictive ones with non-zero coefficients (Figures 3A, B). By means of 10-fold cross-validation, the optimal λ value, which was the most appropriate for the model, was selected. While ensuring the goodness of fit, the fewest variables were included. Finally, lambda. min (λ = 0.032) was chosen as the optimal λ value, and 6 predictive variables with non-zero coefficients were screened out: gender, nasogastric intubation, admission WBC, postoperative NLR, postoperative PLR, and PNI.

#### 3.3.2 Development of the prediction model

**FIGURE 4:**
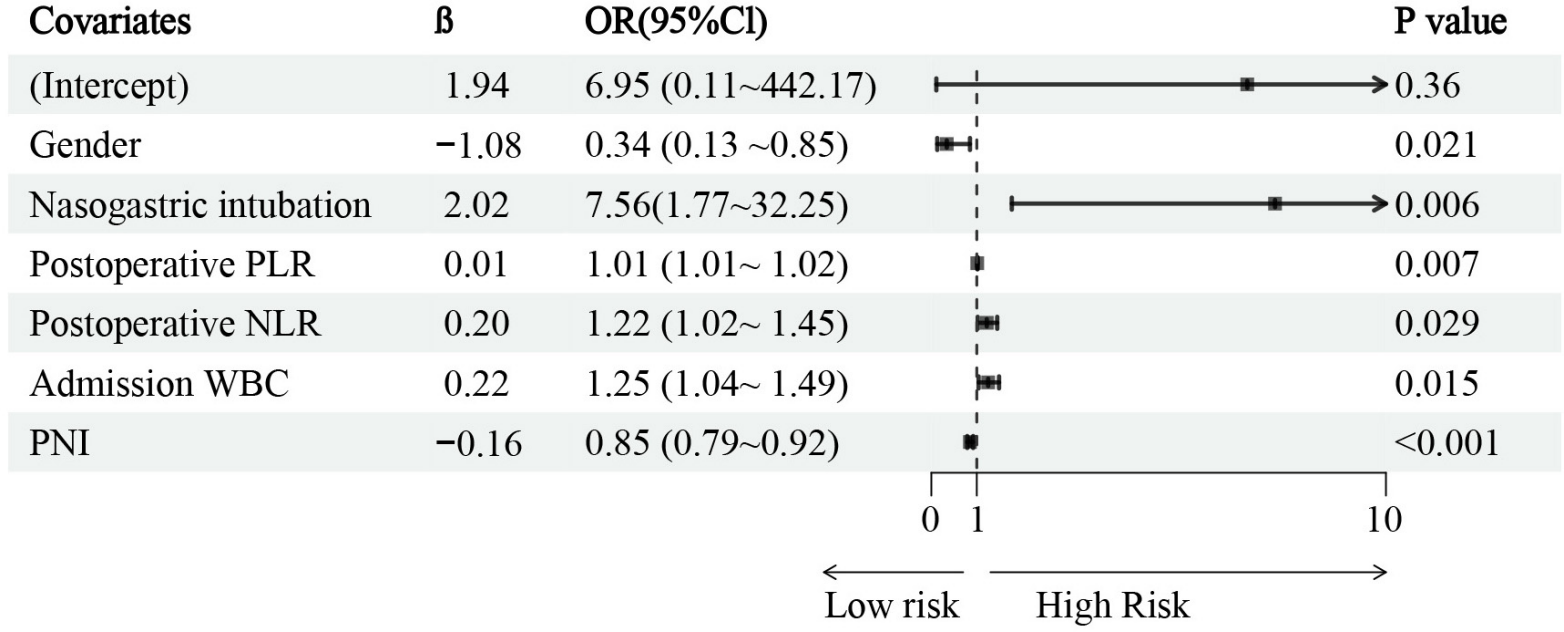
Results of multivariable logistic regression of ventilator-associated pneumonia (VAP) after endovascular therapy (EVT).

**FIGURE 5:**
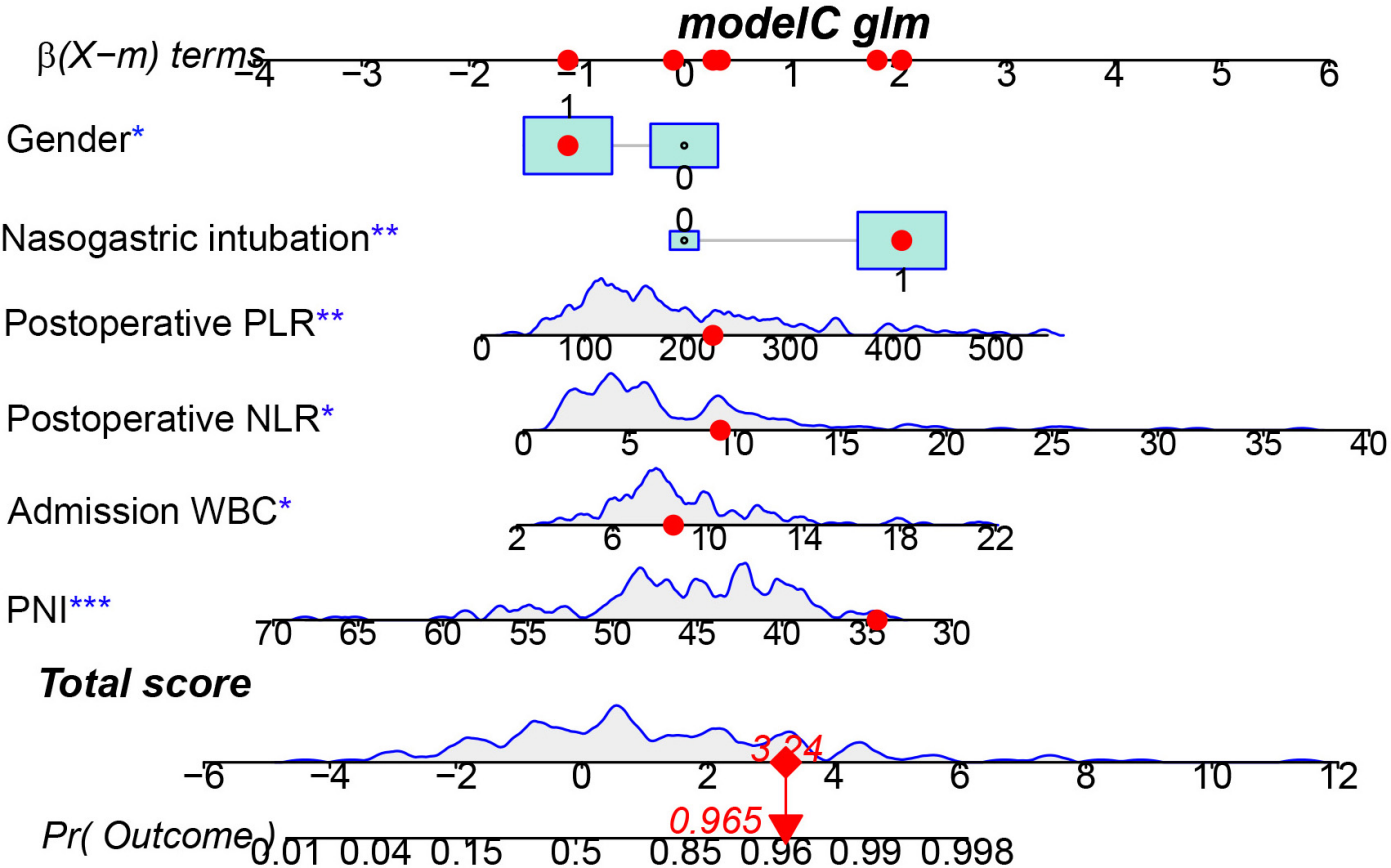
Nomogram predicting the probability of ventilator-associated pneumonia (VAP) in patients after endovascular therapy (EVT) of the training cohort. The dots represent the prediction results of patients. *** represents the highest level of significance (***p < 0.001), followed by ** (** 0.001 < *p* < 0.05), and * (*p < 0.05).

Multivariate logistic regression analysis was conducted on the 7 variables identified by LASSO, with findings indicating that gender, nasogastric intubation, admission WBC, postoperative NLR, âpostoperative PLR, and PNI served as independent risk factors for VAP after EVT (Figure 4, specific data in Supplementary Material 3). All six variables showed statistically significant differences. Consequently, a nomogram for predicting post-stroke VAP was constructed based on these four factors (Figure 5).

### 3.4 Evaluation and clinical application of nomogram

**FIGURE 6:**
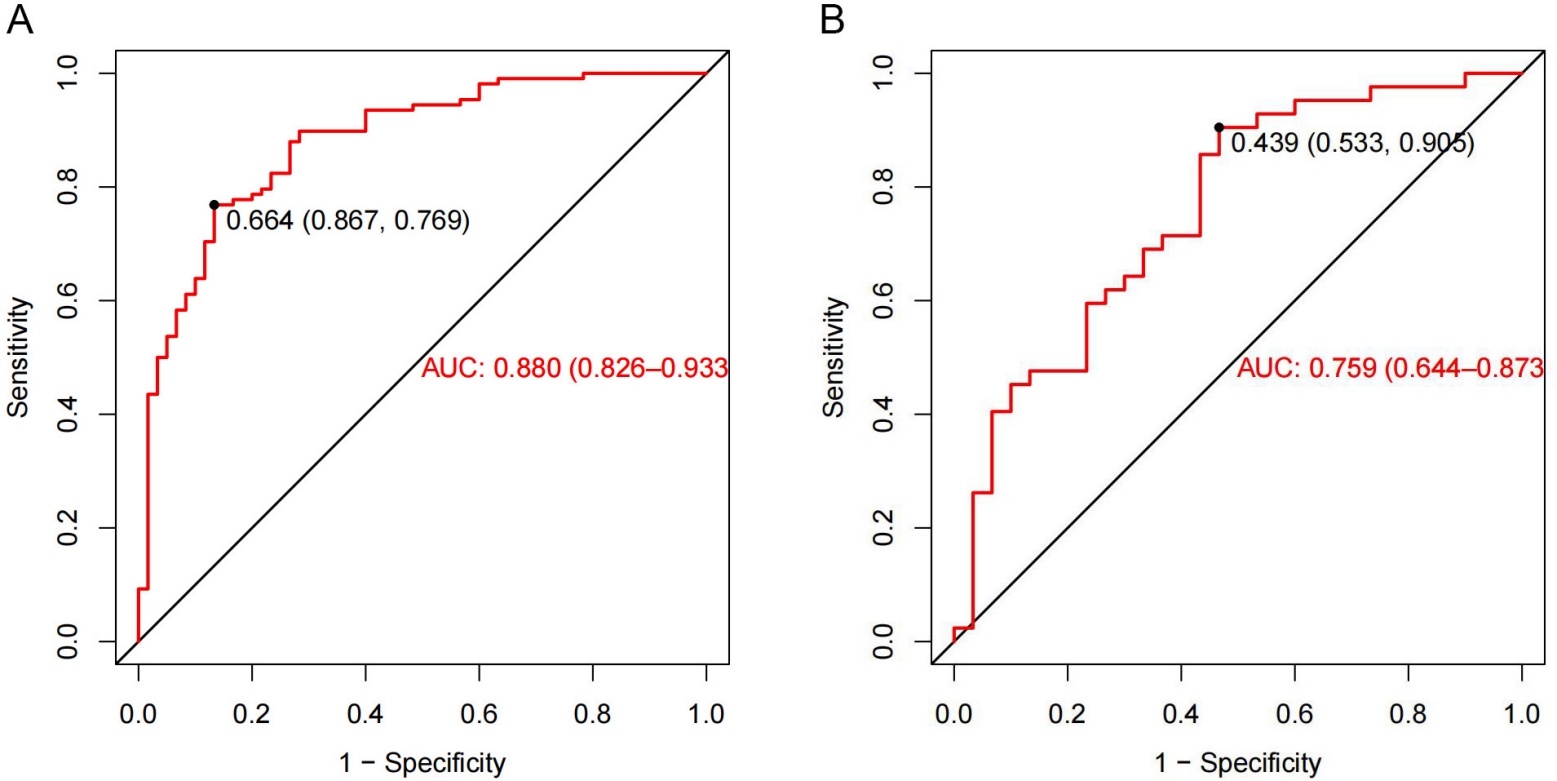
Receiver operating characteristic (ROC) curves for the ventilator-associated pneumonia (VAP) predictive nomogram in acute ischemic stroke with large vessel occlusion (AIS-LVO) patients after endovascular therapy (EVT). In the training cohort **(A)**, the area under the AUROC was 0.880 (95% CI: 0.826–0.933). At a risk probability cut-off of 0.664, the model showed a specificity of 0.867 and sensitivity of 0.769. For the validation cohort **(B)**, the AUROC was 0.759 (95% CI: 0.644–0.873). A threshold of 0.439 yielded a specificity of 0.533 and a sensitivity of 0.905.

**FIGURE 7:**
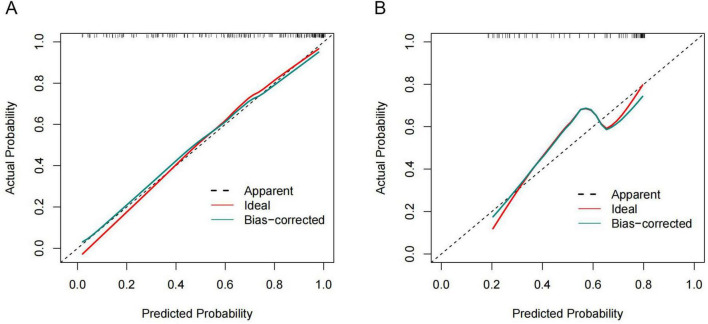
The calibration curves for the VAP nomogram in AIS-LVO patients after EVT. **(A)** Calibration curve for the training cohort. **(B)** Calibration curve for the validation cohort. The diagonal dashed line denotes the benchmark prediction of a perfectly calibrated model, and the solid line represents the probability forecasts derived from the nomogram. The level of congruence between predicted and real outcomes is evaluated by the closeness of the solid line to the dashed line; minimal separation indicates superior calibration performance.

The ROC curve was employed to assess the predictive performance of the model. In the training cohort, the AUROC was 0.880 (95% CI: 0.826–0.933), while in the validation cohort, it was 0.759 (95% CI: 0.644–0.873) (Figures 6A, B), suggesting the nomogram possessed good predictive efficacy. The Hosmer-Lemeshow goodness-of-fit test was utilized for model calibration, and the calibration curves revealed a high degree of concordance between predicted probabilities and actual incidences of post-stroke VAP in both cohorts (Figures 7A, B).

**FIGURE 8:**
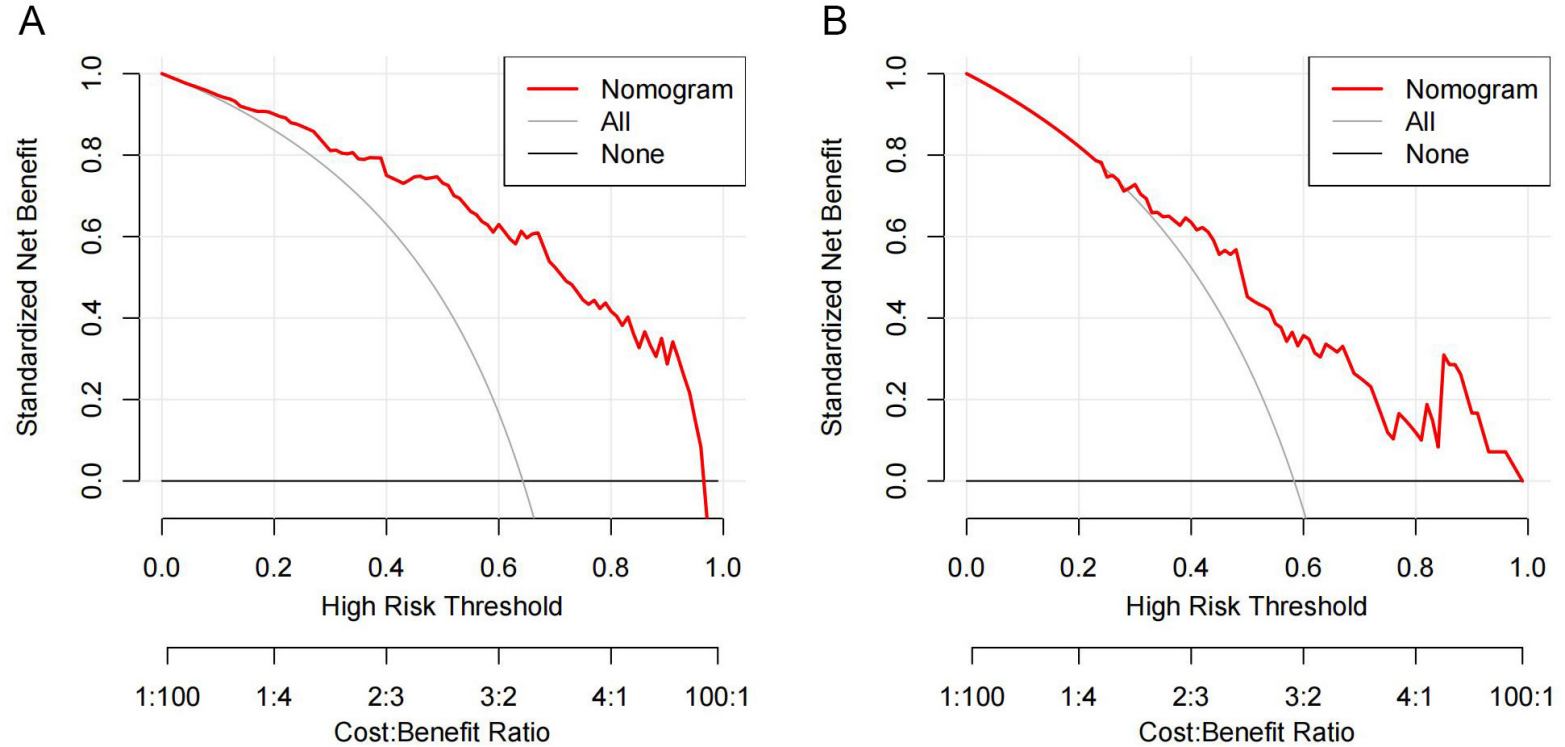
The DCA curves illustrate the performance of the VAP prognostic nomogram in AIS-LVO patients following EVT. **(A)** DCA for the training cohort. **(B)** DCA for the validation cohort. The vertical axis quantifies the net clinical benefit associated with the nomogram. The red curve delineates the VAP clinical diagnostic model for these patients, with the black horizontal line (“no intervention” strategy) and the gray diagonal line (“full intervention” strategy) representing the two polar opposite clinical decision strategies.

**FIGURE 9:**
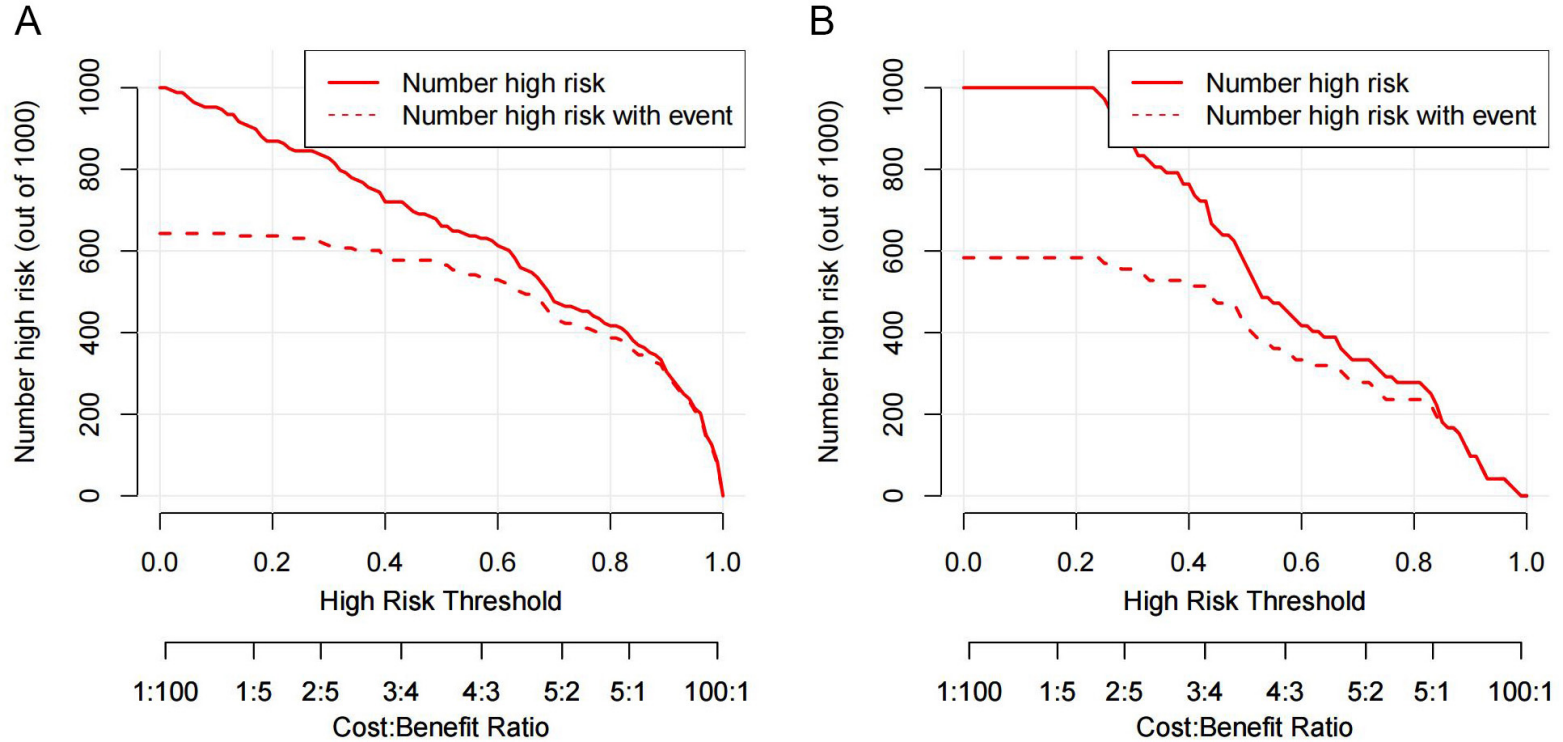
The CIC analysis for the VAP predictive nomogram in AIS-LVO patients is illustrated. **(A)** CIC for the training cohort. **(B)** CIC for the validation cohort. The y-axis enumerates the population at risk for VAP. The solid red line charts the predicted post-stroke VAP episode rate by the model, while the dashed red line marks the measured number of actual VAP cases.

The DCA was utilized to assess the clinical practicality of the nomogram. The generated DCA curves indicated that the model offered substantial net clinical advantages across an appropriate threshold probability spectrum in both the training and validation cohorts (Figures 8A, B). Subsequently, the CIC was developed to further explore and evaluate the model’s clinical value (Figures 9A, B).

## 4 Discussion

The accelerated pace of global population aging has led to a marked increase in stroke incidence among the elderly. This trend is imposing growing strains on public health systems worldwide (Howard and Goff, 2012). Although EVT has dramatically improved revascularization outcomes, postoperative VAP remains a significant complication. It is associated with prolonged hospital stays, increased healthcare costs, and worse neurological recovery (de Jonge et al., 2020; Herpich and Rincon, 2020; Widimsky et al., 2023). This study developed and validated the first predictive model for VAP in elderly AIS-LVO patients treated with EVT, employing rigorous statistical methods. This work addresses a critical gap in the field and provides clinicians with a practical tool for early risk identification.

Clinical investigations have shown that VAP incidence in elderly patients is much greater than in younger patients (Scislo et al., 2022). In this study, the VAP incidence in elderly AIS-LVO patients treated with EVT was as high as 62.2%, a rate that substantially exceeds those in most previous reports. This study’s population characteristics may explain this discrepancy. This likely reflects our focus on elderly patients who typically had poor baseline conditions and various comorbidities like digestive disorders and chronic obstructive pulmonary disease (COPD), both known to independently increase the risk and severity of VAP (Liu and Guo, 2018; Xu et al., 2019). Furthermore, elderly AIS-LVO patients after EVT were in critical condition, which was closely related to the higher incidence of VAP. Additionally, AIS-LVO has been shown to result in neurological impairments, such as impaired consciousness and dysphagia, which in turn can directly increase pneumonia risk. Conversely, existing research indicates that the occurrence of VAP in elderly patients is further exacerbated by several factors, including ICU admission, the method and duration of mechanical ventilation, central venous catheterization, prolonged catheterization, antibiotics administered, and prior use of corticosteroids (Xu et al., 2019). Further investigation is needed to elucidate the mechanisms and potential interventions to mitigate VAP risk in elderly patients.

Our research team previously developed a prediction model for VAP in AIS-LVO patients after EVT based on single-center cohort data (*n* = 184) (Zhu et al., 2024). The nomogram, including GCS score, ICU stay duration, dysphagia, Fazekas scale 2, and admission diastolic blood pressure, can help clinicians identify high-risk patients for VAP in AIS-LVO patients after EVT. Based on prior research, this study further focused on elderly AIS-LVO patients after EVT to carry out an in-depth analysis of VAP risk factors. Through multivariate regression analysis, we constructed a nomogram and identified six independent predictive factors for VAP, which reflect the multifactorial nature of VAP pathogenesis. These encompass invasive procedures (e.g., nasogastric intubation), systemic inflammation (postoperative NLR, PLR, and admission WBC), and immune-nutritional status (PNI). A key innovation of our model is the incorporation of composite indicators reflecting systemic inflammation (NLR, PLR) and immune-nutritional status (PNI). This moves beyond conventional clinical factors like age and NIHSS score, adding a new dimension for early identification of high-risk VAP patients.

We conducted a three-month follow-up survey of VAP patients. The results revealed that these patients had worse neurological outcomes and higher mortality rates than those without pulmonary infection. The results suggested that gender serves as an independent risk factor for VAP in elderly AIS-LVO patients after EVT. Consistent with previous studies, there is a higher occurrence of VAP in male patients compared to female patients (Sharpe et al., 2014). However, the mortality rate associated with VAP is notably higher in female patients (Sharpe et al., 2014). The present study aligns with these earlier observations, as the total number of fatalities during the study’s follow-up period was 22. Of these, 16 deaths occurred among female patients, constituting 72.7% of the total mortality count.

Long-term nasogastric intubation, as an invasive procedure, can compromise the body’s immune system, disrupting the delicate microecological balance within the oropharynx and stomach. This allows pathogens to migrate and colonize the lower respiratory tract, thereby increasing the risk of infection. Clinical data demonstrate that patients with indwelling gastric tubes exhibit a 3–5 times higher risk of developing gastric reflux and aspiration compared to patients without gastric tubes (Xu et al., 2021). The pathogenic bacteria carried by the refluxed material can invade the lungs directly (Xu et al., 2021), which may serve as a crucial factor in the triggering of VAP. Furthermore, elevated leukocyte levels at admission mark a state of systemic inflammation and have significant predictive value for the onset of postoperative pneumonia. A high WBC count indicates that the patient’s immune system is mounting an aggressive response to infection preoperatively (Doganci et al., 2024; Nelde et al., 2024), thereby making them susceptible to postoperative complications like pneumonia.

This study is the first to demonstrate postoperative NLR and PLR as independent indicators for VAP risk in elderly AIS-LVO patients after EVT, offering a new perspective for preventing and treating post-stroke infection using composite index markers. Prior research indicated that NLR and PLR, as novel inflammatory markers, exhibit a strong association with poor prognosis following mechanical thrombectomy in individuals with acute ischemic stroke. Elevated levels of these markers frequently indicate increased stroke severity (Han et al., 2023; Yi et al., 2021). This finding establishes a theoretical foundation for the present study. From the perspective of pathophysiological mechanisms, following the occurrence of acute cerebral infarction, brain tissue injury rapidly activates systemic inflammatory response syndrome (SIRS). On the one hand, the rapid process of neutrophil activation and the subsequent release of cytokines is accelerated, while lymphocytes, due to their immunosuppressive state, result in a substantial elevation in the NLR (Li et al., 2023). On the other hand, platelet activation and impaired lymphocyte function are associated. This study found significantly higher NLR levels in patients who developed pneumonia compared to those who did not contract the infection, supporting the idea that NLR is a reliable marker for systemic inflammation and a potential predictor for pneumonia in stroke patients. PLR is essential in the processes of inflammation and immune imbalance. Platelets can regulate inflammation by releasing cytokines and growth factors in response to inflammatory stimulation. Furthermore, infection-induced lymphocyte dysfunction and apoptosis contribute to a considerable increase in PLR in VAP patients (Li and He, 2022; Yu et al., 2022; Zawiah et al., 2023). The study data further confirmed that VAP patients had considerably greater NLR and PLR levels than non-VAP patients. Both the NLR and PLR were identified as independent risk indicators of VAP development. The clinical application of the NLR and the PLR offers distinct advantages: these ratios are derived from routine blood tests, making them cost-effective and readily available. Their rapid calculation facilitates the early identification of high-risk patients, enabling timely intervention and potentially improving outcomes.

The Prognostic Nutritional Index (PNI) can quantify integrated immune-nutritional status by integrating lymphocyte counts and serum albumin levels (Onodera et al., 1984). This study revealed that a lower PNI was substantially correlated with an increased risk of VAP. Malnutrition can increase VAP risk through two primary mechanisms. First, it can directly reduce the T-cell populations in patients, resulting in impaired cellular immune function (Zhang et al., 2021). Second, it can weaken phagocyte activity, interfere with the synthesis of complement, and thus reduce the body’s ability to eliminate pathogens and resist viruses (Chandra, 1997). Furthermore, nutritional deficiencies can impede muscle tissue regeneration and energy provision, consequently causing respiratory muscle weakness or atrophy. This leads to an impaired cough and respiratory distress. (Marcos et al., 2003). This, in turn, can further increase the risk of pneumonia. A multitude of clinical investigations have proved the efficacy of PNI in disease prediction, such as evaluating the severity of patients with coronavirus disease 2019 (COVID-19) (Ekinci et al., 2022; Xue et al., 2020), predicting the prognosis of patients with chronic obstructive pulmonary disease (COPD) (Suzuki et al., 2023), and assessing mortality risk in patients with lung cancer and community-acquired pneumonia (Bahçeci et al., 2022). To our knowledge, this study is the first to report an independent association between low PNI and increased VAP risk in elderly AIS-LVO patients following EVT. Consequently, prompt nutritional support is recommended for patients with a low PNI. Providing high-quality protein and essential nutrients can enhance patients’ immune defenses, ultimately reducing VAP incidence and improving stroke prognosis.

Basing our exploration on this foundation, a comprehensive examination of risk factors and the development of a prediction model offer significant theoretical value and clinical significance for reducing infection risk and enhancing stroke patients’ prognosis.

## 5 Conclusion

In elderly patients, acute ischemic stroke with large vessel occlusion (AIS-LVO) results in significant disability and mortality; post-stroke ventilator-associated pneumonia (VAP) compounds this burden by impairing neurological recovery and worsening clinical outcomes. This study constructed and validated a nomogram prediction model based on the risk factors for VAP in elderly AIS-LVO after EVT. The results indicated that gender, the presence of an indwelling gastric tube, admission leukocyte level, postoperative NLR, postoperative PLR, and PNI were independent predictors of VAP. The model has potential for utilization as an effective instrument in the early identification of high-risk patients.

## 6 Limitations

This study still has several limitations. Firstly, the retrospective design may limit the generalizability of our findings to current patient populations. Secondly, the oral health assessment of hospitalized patients, which is highly associated with pneumonia risk, has not been thoroughly investigated (Awano et al., 2008; Son et al., 2020). Thirdly, the critical condition of most patients, many of whom required continuous mechanical ventilation, precluded the performance of pulmonary function tests. In fact, some patients exhibited unstable vital signs and impaired consciousness, which rendered them unable to actively cooperate with the pulmonary function tests, such as forceful exhalation and inhalation. Furthermore, the separation of patients from ventilators during the tests might cause serious complications, such as hypoxemia, resulting in a lack of pulmonary function tests. Fourthly, incomplete data in some cases, partly due to updates in the electronic medical record system, represents a limitation. Additionally, the study population included patients with COPD; however, patients with other comorbid lung diseases (such as bronchiectasis) were not included in the analysis, thereby limiting the comprehensiveness of the data. Furthermore, the temporal scope of the study and the single-center design have resulted in an inadequate representation of patient diversity, which may have influenced the model’s prediction accuracy. Future studies may be optimized for improvement by including multicenter data and exploring more influential variables.

## Data Availability

The raw data supporting the conclusions of this article will be made available by the authors, without undue reservation.
