# Supplementary material for: Development and validation of a prognostic nomogram for predicting ventilator-associated pneumonia risk in elderly large vessel occlusion ischemic stroke after endovascular therapy patients
Source: Front Aging Neurosci. 2026 Jan 8;17:1654146. doi: 10.3389/fnagi.2025.1654146 (PMC12823881; doi:10.3389/fnagi.2025.1654146)
Supplement: Supplementary file 3 [file Table_3.docx]

*Statistical Power Analysis*

*VAP Prediction Study in Acute Ischemic Stroke Patients*

To verify the adequacy of sample size for detecting intergroup differences, a post-hoc power analysis was performed using G*Power 3.1 software. Based on actual data from the VAP group (n=150) and non-VAP group (n=90), Cohen's d effect sizes of key predictors (e.g., platelet-to-lymphocyte ratio, systemic immune-inflammatory index) were calculated, with a mean Cohen's d=0.80 (medium-to-large effect size). Setting parameters as effect size d=0.80, α=0.05 (two-tailed), and sample sizes n1=90 (non-VAP) and n2=150 (VAP), the two independent samples t-test showed a statistical power of 99.99% (1-β=0.99997), substantially exceeding the recommended threshold of 80%. This confirmed that the sample size was sufficient to support the detection of intergroup differences.

#### G * Power Analysis Results

### 1.1 Analysis Parameter Settings

| **Parameters** | **Set value** |
| --- | --- |
| Test family | t tests |
| Statistical test | Means: Difference between two independent means |
| Type of power analysis | Post hoc: Compute achieved power |
| Tail(s) | Two (two-sided test) |
| **Effect size d** | **0.8** |
| α err prob | 0.05 |
| Sample size group 1 (Non-VAP) | 90 |
| Sample size group 2 (VAP) | 150 |

### 1.2 Calculation results

| output parameter | Calculated value |
| --- | --- |
| Noncentrality parameter δ | 6.000000 |
| Critical t | 1.9699815 |
| Df (degree of freedom) | 238 |
| **Power (1-β err prob)** | **0.9999704 (>99.99%)** |

**结论：**在α=0.05、效应量d=0.8、样本量n=240（Non-VAP:90, VAP:150）的条件下，本研究达到的统计效能为99.99%（1-β=0.9999704），远超推荐的80%阈值，表明样本量高度充足。Conclusion: Under the conditions of α=0.05, effect size d=0.8, and sample size n=240 (Non-VAP:90, VAP:150), this study achieved a statistical power of 99.99% (1-β=0.9999704), far exceeding the recommended 80% threshold. This indicates that the sample size is highly adequate.

#### 2.效应量计算依据The calculation basis of effect quantity

基于研究实际数据计算的关键炎症生物标志物效应量：The effect size of key inflammatory biomarkers calculated based on the actual data of the study :

| **Variable** | **Cohen's d** | **Classification** | **P值** |
| --- | --- | --- | --- |
| PLR (training sets) | **1.001** | **Large** | <0.001 |
| SII (training sets) | **0.899** | **Large** | <0.001 |
| NLR (training sets) | **0.843** | **Large** | <0.001 |
| PNI (training sets) | 0.630 | Medium | <0.001 |
| **Average Effect Size** | **0.80** | **Large** |  |

**注：**Cohen's d标准：Small (0.2-0.5), Medium (0.5-0.8), Large (≥0.8). PLR=血小板/淋巴细胞比值, SII=系统免疫炎症指数, NLR=中性粒细胞/淋巴细胞比值, PNI=预后营养指数

Note: Cohen's d standards: Small (0.2–0.5), Medium (0.5–0.8), Large (≥0.8). PLR = Platelet-to-lymphocyte ratio, SII = Systemic Inflammatory Index, NLR = Neutrophil-to-lymphocyte ratio, PNI = Prognostic Nutritional Index
